# Comparative Evaluation of Redox and Non-Redox Epoxy–Clay Coatings for Corrosion Resistance in ACQ Saline Media

**DOI:** 10.3390/polym17121684

**Published:** 2025-06-17

**Authors:** Yun-Xiang Lan, Yun-Hsuan Chen, Hsin-Yu Chang, Karen S. Santiago, Li-Yun Su, Cheng-Yu Tsai, Chun-Hung Huang, Jui-Ming Yeh

**Affiliations:** 1 Department of Chemistry, Center for Nanotechnology at Chung Yuan Christian University, Chung Li 320314, Taiwan; aa123451014@gmail.com (Y.-X.L.); miffy7769@gmail.com (Y.-H.C.); b0966155770@gmail.com (H.-Y.C.); 2 Department of Chemistry, College of Science, Research Center for the Natural and Applied Sciences, University of Santo Tomas, Manila 1015, Philippines; kssantiago@ust.edu.ph; 3 Department of Chemical and Materials Engineering, Southern Taiwan University of Science and Technology, Tainan 710301, Taiwan; 4 Ruei-Tsan Industrial Co., Ltd., Kaohsiung City 821007, Taiwan; jenu168@gmail.com (C.-Y.T.); jinhong0808@gmail.com (C.-H.H.)

**Keywords:** aniline oligomer, clay, corrosion, nanocomposite, ACQ

## Abstract

This study prepared epoxy–clay nanocomposites (ECNs) by incorporating organophilic clays modified with either non-redox cetyltrimethylammonium bromide (CTAB) or redox-active aniline pentamer (AP), then compared their anticorrosion performance on metal substrates in saline environments. The test solution contained 2 wt% alkaline copper quaternary (ACQ) wood preservatives. Cold-rolled steel (CRS) panels coated with the ECNs were evaluated via electrochemical impedance spectroscopy (EIS) in saline media both with and without ACQ. For CRS coated with unmodified epoxy, the Nyquist plot showed impedance dropping from 255 kΩ to 121 kΩ upon adding 2 wt% ACQ—indicating that Cu^2^⁺ ions accelerate iron oxidation. Introducing 1 wt% CTAB–clay into the epoxy increased impedance from 121 kΩ to 271 kΩ, while 1 wt% AP–clay raised it to 702 kΩ. This improvement arises because the organophilic clay platelets create a more tortuous path for Cu^2+^ and O₂ diffusion, as confirmed by ICP–MS measurements of Cu^2+^ after EIS and oxygen permeability tests (GPA), thereby slowing iron oxidation. Moreover, ECN coatings containing AP–clay outperformed those with CTAB–clay in corrosion resistance, suggesting that AP not only enhances platelet dispersion but also promotes formation of a dense, passive metal oxide layer at the coating–metal interface, as shown by TEM, GPA, and XRD analyses. Finally, accelerated salt-spray exposure following ASTM B-117 yielded corrosion behavior consistent with the EIS results.

## 1. Introduction

Wood offers distinct advantages as a building material, with its natural grain, rich color variations, and pleasing aroma providing a unique aesthetic. As a renewable and recyclable resource, wood occupies a special place in construction due to its lightweight nature, high strength, ease of processing, and low waste production. This combination of durability and beauty makes it a popular choice for construction in countries including Japan and the United States, where timber structures are widely utilized, showcasing wood’s versatility in building applications. However, wood is susceptible to biological degradation by fungi, termites, and other organisms [1,2,3]. As a result, various methods have been developed over the years to enhance wood’s durability.

Chemical preservatives continue to be among the most commonly utilized and effective means for safeguarding wood. To extend the durability of outdoor wood structures, water-based formulations containing metallic or organometallic salts are routinely employed. These treatments are designed to prevent biodegradation such as rot, mold, and insect infestation. In Canadian residential construction, wood is frequently treated with copper-based preservatives, including copper azole (CA) and alkaline copper quaternary (ACQ) ammonium. ACQ typically comprises 67% copper oxide and 33% quaternary ammonium compounds, and is available in multiple commercial variants [4]. ACQ Type D, formulated with monoethanolamine (C_2_H_7_NO), is commonly applied to softwood species, whereas ACQ Type B, which utilizes ammonia (NH_3_), is generally reserved for wood that is more resistant to preservative uptake [1]. Despite their protective intent, studies have shown that these waterborne preservatives can increase the probability of metal corrosion when in contact with treated lumber [5,6,7,8,9,10,11,12,13]. It is estimated that more than 95% of the copper in preserved wood exists in the ionic Cu^2^⁺ form, which becomes electrochemically reactive when it interfaces with steel or galvanized fasteners, primarily due to thermodynamic factors [12]. Experiments have demonstrated that corrosion rates of metal fasteners embedded in treated wood escalate in tandem with rising Cu^2^⁺ concentrations, suggesting that these ions play an active role in accelerating the corrosion process [11]. It has been further hypothesized that Cu^2+^ ions migrate toward the metal surface and undergo reduction, thereby perpetuating the corrosion mechanism. Therefore, appropriate preventive measures are essential when employing aqueous-based preservatives to limit the degradation of embedded metallic components [14]. The concern surrounding “elevated metal corrosion rates in contact with treated wood” has thus emerged as a significant issue for wood-related applications [11,14,15,16,17,18].

Montmorillonite (MMT) is a 2D silicate clay mineral characterized by its layered morphology, where individual silicate platelets—each approximately 10 Å thick and 2180 Å long—are stacked, resulting in a high aspect ratio of around 220 [19,20,21,22,23]. Structurally, each platelet consists of a sandwich-like configuration: two tetrahedral silica sheets enveloping a central octahedral layer composed of magnesium or aluminum hydroxides [24,25]. The interlayer space harbors exchangeable inorganic cations (e.g., Na^+^, Ca^2+^), which can undergo ion exchange with organic cations, enabling the transformation of MMT into an organophilic variant known as organically modified clay (OMT) [26]. Owing to its notable swelling capacity, MMT is particularly suitable for intercalation with polymer matrices. This layered configuration facilitates enhancements in thermal stability, mechanical strength, flame resistance, and impermeability to small molecules. Prior research has shown that MMT can impede the penetration of moisture and oxygen, thereby forming an effective corrosion-inhibiting barrier for metallic substrates [19,27,28,29,30]. Previous investigations have shown that the incorporation of organically modified clays (OMCs) into various polymer matrices can significantly enhance the anticorrosive performance of the resulting composites. These improvements have been observed in both conjugated systems, such as polyaniline [27,31] and polypyrrole [32], and in conventional non-conjugated polymers, including polymethyl methacrylate [33] and polystyrene [34,35,36]. The enhanced protective behavior is primarily attributed to the barrier effect provided by well-dispersed clay platelets, which obstruct the ingress of corrosive species. Despite these advances, the specific role of OMC in impeding copper ion diffusion remains insufficiently clarified, particularly in coatings applied to metallic substrates treated with copper-based wood preservatives such as alkaline copper quaternary (ACQ). In a more recent study, Yeh et al. demonstrated that uniform dispersion of OMC within epoxy matrices notably improves corrosion resistance under both saline conditions and environments containing ACQ-derived Cu^2+^ species [37]. These findings underscore the potential of OMC-filled epoxy systems in the design of protective coatings for chemically aggressive applications, although further investigation is still needed to fully elucidate the Cu^2+^ inhibition mechanism at the molecular level.

In the past decades, Wei et al. has focused on the synthesis of electroactive aniline oligomers of well-defined structures [38]. Since the end group of aniline oligomers can be readily varied to polymerizable functionalities, the oligomers function as monomers for further polymerization to yield a variety of new polymers, which possess both excellent mechanical properties of typical polymers and the electroactivity of the conducting polymers to a certain extent. For example, Yeh et al. reported that the preparation and electro-optical display device application of liquid crystal composite materials containing aniline pentamer modified clay [39]. Yeh et al. reported the dopable polyimide membrane containing amine-terminated aniline trimer (ACAT) with excellent gas separation capability [40]. Moreover, Ding et al. reported that incorporating 1.0 wt% aniline tetramer (AT), synthesized via chemical oxidation, into epoxy coatings significantly enhances corrosion protection, owing to AT’s redox activity, adsorption capability, and synergistic curing behavior with polyamide, as confirmed by EIS and polarization analyses [41].

Therefore, for this paper, we prepared a series of ECN coatings, which contained intercalating agents of non-redox CTAB and redox AP and comparatively investigated their corrosion protection in distinctive metallic substrates in a saline environment without and with ACQ. First, the AP was synthesized by oxidative coupling reaction, followed with characterization by ^1^H-NMR, FTIR, and mass spectroscopy. Redox capability of AP was investigated by electrochemical cyclic voltammetry and monitored by UV–visible spectroscopy. Subsequently, the OMCs were prepared by performing the cationic exchange reaction between the intercalating agent (i.e., of non-redox CTAB and redox AP) and Na^+^-MMT clay. The OMCs, denoted by CTAB-clay and AP-clay, were subsequently characterized by FTIR, XRD pattern, and TGA. Eventually, a series of ECN coatings containing 1 and 5 wt% of OMCs were prepared by dispersing clay platelets into epoxy coating (denoted by CTAB-clay-1, CTAB-clay-5, AP-clay-1, and AP-clay-5, respectively). The ECN materials were subsequently characterized by FTIR spectra. The dispersion capability of OMC platelets in ECN materials was investigated by TEM. Surface wettability of ECN materials was determined by the contact angle of water droplets. Corrosion protection of the ECN coatings upon CRS electrode was determined by EIS studies. Accelerated salt-spray tests of ECN coatings upon steel panels in an aggressive medium were also evaluated based on the ASTM B-117.

## 2. Experimental Section

### 2.1. Chemicals and Instrumentation

All reagents were used as received without additional purification. Diglycidyl ether of bisphenol A (DGEBA; Aldrich, Burlington, MA, USA; 95.0%) served as the epoxy resin, and trimethylolpropane tris(poly(propylene glycol), amine-terminated) ether (T-403; Mw 440; Aldrich, Burlington, MA, USA; 97.0%) was employed as the curing agent. Montmorillonite clay (Nanocor; cation exchange capacity 120 meq/100 g) and alkaline copper quaternary (ACQ; provided by Ruei-Tsan Industrial Co., Ltd. KHH, Taipei, Taiwan) were incorporated directly. 4,4′-Diaminodiphenylamine sulfate (TCI, Chuo-ku, Tokyo, Japan; 97.0%) and N,N′-diphenyl-p-phenylenediamine (Aldrich, Burlington, MA, USA; 98%) were used as received as redox-active additives. N,N-Dimethylformamide (DMF; Avantor™, Phillipsburg, NJ, USA; 99.9%) and N,N-dimethylacetamide (DMAc; Avantor™, Phillipsburg, NJ, USA; 99.9%) served as solvents for clay modification. Sodium chloride (NaCl; Aldrich, Burlington, MA, USA; 99.0%), hydrochloric acid (HCl; Aldrich, Burlington, MA, USA; 37.0%), ammonium hydroxide solution (NH_4_OH; Aldrich, Burlington, MA, USA; 28.0–30.0%), and ammonium persulfate (APS; Avantor™, Phillipsburg, NJ, USA; 97.0%) were employed in the preparation of modified clay and characterization procedures. Except where otherwise indicated, all other chemicals and solvents were of analytical grade and used without further treatment.

Proton nuclear magnetic resonance (^1^H-NMR) spectra were acquired on a Bruker 300 MHz spectrometer, with tetramethylsilane (TMS) serving as the internal chemical shift reference. Deuterated dimethyl sulfoxide (DMSO-d_6_) was employed as the solvent for all NMR measurements. Fourier transform infrared (FTIR) spectra in attenuated total reflectance (ATR) mode were recorded on a JASCO 4200 instrument (Hachioji, Tokyo, Japan) at ambient temperature, scanning from 4000 to 400 cm⁻^1^ with a spectral resolution of 4.0 cm⁻^1^. Mass spectrometric analyses were performed on a Bruker Daltonics Esquire 2000 ion trap mass spectrometer (Leipzig, Germany), equipped with an Agilent ESI source (model G1607-6001) (Santa Clara, CA, USA). The nanoscale morphology of the epoxy–clay nanocomposite (ECN) samples was examined by transmission electron microscopy (TEM) using a JEOL-200FX microscope (Akishima, Tokyo, Japan). Prior to imaging, ultrathin sections (60–90 nm) were prepared with a diamond knife. Wide-angle X-ray diffraction (XRD) patterns were collected on a Rigaku D/MAX-3C OD-2988N diffractometer (Tokyo, Japan) employing a copper target and nickel filter, with a scan rate of 4° per minute. Thermogravimetric analysis (TGA) was conducted on a TA Instruments TGA-Q50 (New Castle, DE, U.S.) under a nitrogen atmosphere; samples were heated from 30 °C to 800 °C at a rate of 10 °C min⁻^1^ to assess both composition and thermal stability. Ultraviolet–visible (UV–vis) absorption spectra were recorded on a Hitachi U-2000 spectrophotometer (Chiyoda-ku, Tokyo, Japan). Surface wettability of the coatings was evaluated by measuring the static water contact angle (CA) at room temperature using a First Ten Angstroms FTA-125 goniometer (Portsmouth, VA, USA). Oxygen permeability measurements were carried out with a GTR10-GPA gas permeability analyzer (Yanagimoto Co. Ltd., Takaishi -shi, Osaka, Japan). To quantify copper ion concentration in ACQ solutions before and after electrochemical impedance spectroscopy (EIS) tests, inductively coupled plasma mass spectrometry (ICP-MS) was utilized. Electrochemical corrosion behavior of the coatings was investigated using a VoltaLab 40 potentiostat/galvanostat (Yanagimoto Co. Ltd., Takaishi -shi, Osaka, Japan) in a conventional three-electrode cell containing a graphite rod as the counter electrode, a saturated calomel electrode (SCE) as the reference, and the coated cold-rolled steel (CRS) panel as the working electrode. Accelerated salt-spray corrosion tests were performed in accordance with ASTM B-117 using a salt-spray chamber from Ten Billion Technology Co., Ltd (Tainan, Taiwan). The CRS electrodes used for electrochemical measurements were 18.5 mm in diameter and 1.2 mm in thickness, whereas the steel panels subjected to salt-spray exposure were cut to 150 mm × 70 mm × 0.786 mm.

### 2.2. Synthesis of Aniline Pentamer (AP)

**Scheme 1:**
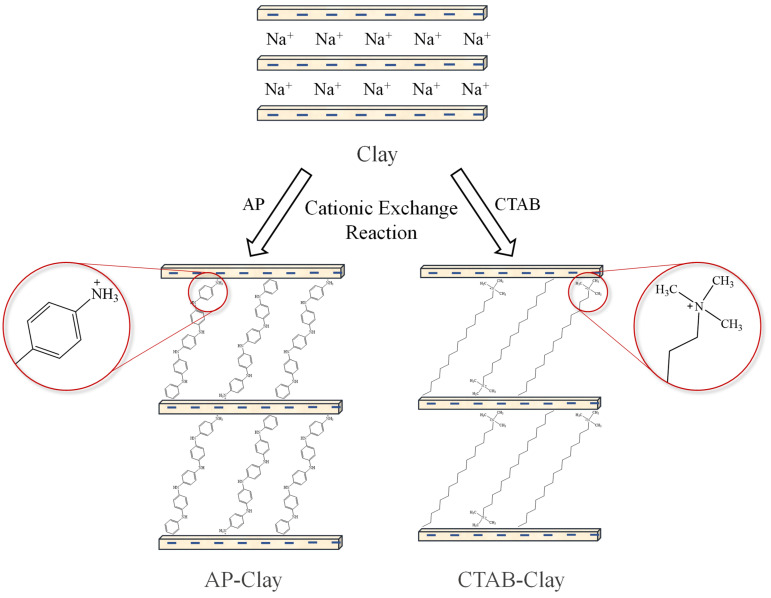
Schematic representation of the synthesis of PGV-AP, and PGV-CTAB.

N,N′-Diphenyl-p-phenylenediamine (2.60 g, 10 mmol) and 4,4′-diaminodiphenylamine sulfate (3.50 g, 10 mmol) were first dissolved in a co-solvent blend consisting of 100 mL DMF, 20 mL deionized water, and 15 mL concentrated HCl. Separately, 2.28 g (10 mmol) of ammonium persulfate (APS) was dissolved in 50 mL of 1.0 N HCl. Under vigorous stirring and with the reaction temperature held at 5 °C, the APS solution was added dropwise to the amine-containing mixture over the course of one hour. A blue precipitate formed during this period, which was subsequently isolated by vacuum filtration. The solid was washed three times with 1.0 N NH_4_OH to remove residual acid and unreacted species, then dried in a vacuum oven at 50 °C for 24 h. The final yield of blue aniline pentamer (AP) was approximately 40% (see Scheme 1).

### 2.3. Organophilic Clay Prepared by Performing the Cationic Exchange Reaction Between Na^+^-MMT Clay and CTAB

**Scheme 2:**
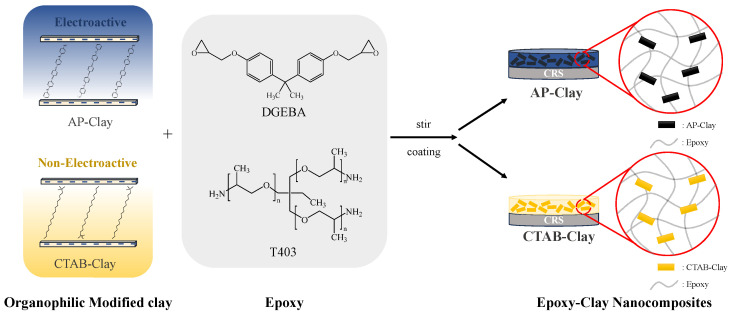
Flowchart for the preparation of CRS electrode coated with distinctive epoxy-clay nanocomposite coatings containing AP-clay, and CTAB-clay.

The 6.0 g of MMT clay (CEC value of 145) was added to 600 mL of deionized water, followed by stirring at 500 rpm at room temperature for 24 h (denoted by beaker A). An amount of 5.9945 g of CTAB was dissolved in 100 mL of deionized water under constant stirring of 500 rpm at room temperature. Acetic acid (1.0 M CH_3_COOH) was added to adjust the pH to ~3–4, then heated to 70 °C and stirred for 30 min (denoted by beaker B). The clay solution of beaker A was slowly added to the modifier solution of beaker B, followed by stirring at 70 °C and 500 rpm for 24 h. After centrifugation, the supernatant was discarded, and the precipitated modified clay was washed with deionized water until neutral. The modified clay was frozen to remove excess water by freeze-drying technique. The resulting powder was ground using an agate mortar to yield organophilic clay powder (denoted CTAB-clay), as shown in Scheme 2.

### 2.4. Organophilic Modified Clay Prepared by Performing the Cationic Exchange Reaction Between Na^+^-MMT Clay and AP

The 1.0 g of MMT clay was added to 30 mL of deionized water, followed by stirring at 500 rpm at room temperature for 24 h (denoted by beaker A). The 0.99082 g of as-synthesized AP was poured into 9.36 mL of H₂SO₄ solution (pH < 1), followed by stirring at 500 rpm at room temperature for 24 h (denoted by beaker B). The solution from beaker A was slowly added into the solution from beaker B at 25 °C in a chilled water bath, then stirred at 600 rpm for 24 h to perform the cationic exchange reaction. The obtained mixture was then centrifuged at 6000 rpm for 30 min. The supernatant was discarded, and the precipitated modified clay was washed with ethanol (C₂H₅OH) and centrifuged repeatedly until the supernatant was neutral and unreacted AP was removed. The modified clay was frozen to remove excess ethanol by freeze-drying technique. The resulting powder was then ground using an agate mortar to yield organophilic clay powder (denoted AP-clay), as shown in Scheme 2.

### 2.5. Fabrication of the CRS Electrode Coated with Distinctive Epoxy Coatings

ECNs containing distinctive OMC (CTAB-clay and AP-clay) were dispersed in 1.5 mL of DMAc, followed by magnetic stirring for 12 h. Subsequently, 1 g of DGEBA was added to the solution, followed by stirring for another 12 h. Afterward, 0.4 g of T-403 was introduced, and the mixture was stirred for an additional 24 h. A suitable amount of the epoxy slurry was applied to CRS electrodes (1 × 1 cm^2^). The coated ECN samples were then placed in an oven for controlled programmed heating, initially at 50 °C for 1 h, followed by curing at 120 °C for 2 h and 140 °C for 4 h. The thickness of distinctive epoxy coatings upon the CRS electrodes was estimated at ~150 μm.

### 2.6. Electrochemical Measurements

Cyclic voltammetry (CV) measurements were carried out in a conventional three-electrode cell, using a saturated calomel electrode (SCE) as the reference and a platinum wire as the counter electrode. The working electrodes consisted of 1.0 × 1.0 cm^2^ platinum foils onto which the active coatings were applied. Scans were recorded from –0.5 V to +0.5 V at a sweep rate of 50 mV s⁻^1^ in 1.0 M H_2_SO_4_, following our earlier protocols and taking advantage of the improved electrochemical stability in acidic media.

**Scheme 3:**
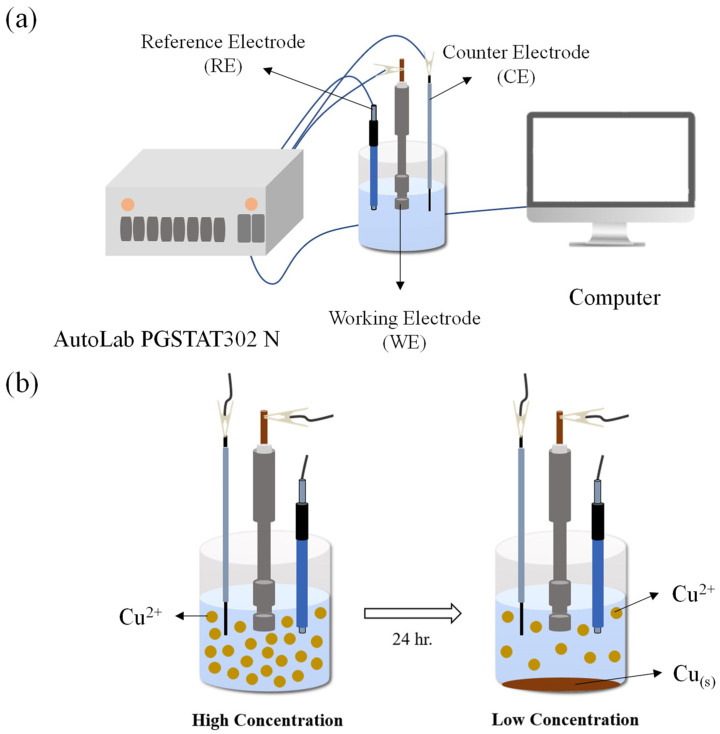
(**a**) Schematic diagram of a three-electrode system device used for electrochemical impedance spectroscopy (EIS) measurements. (**b**) Schematic diagram of corrosion chamber employed before and after EIS testing.

Electrochemical impedance spectroscopy (EIS) was performed on cold-rolled steel (CRS) electrodes (1.0 × 1.0 cm^2^) bearing the respective epoxy coatings. Prior to EIS, each CRS sample was immersed for 2 h in a 3.5 wt% NaCl solution, with or without ACQ, to simulate saline exposure. To prevent electrolyte leakage, the edges of the CRS electrodes were sealed using a high-strength, fast-curing epoxy cement (SPAR^®^). In the three-electrode configuration, the coated CRS served as the working electrode, a graphite rod as the counter, and the SCE as the reference. All coated electrodes were immersed in 3.5 wt% saline (with or without ACQ) during testing. CV data were collected using a VoltaLab PGSTAT204 potentiostat/galvanostat. For EIS, spectra were obtained at open-circuit potential (OCP)—taken as the corrosion potential, Ecorr (mV vs. SCE)—over the frequency range from 10 Hz to 100 MHz. An AutoLab PGSTAT302N (Metrohm/Agnagimoto Co., Ltd., Taipei, Taiwan) was employed to record the impedance response. The full electrochemical setup is depicted in Scheme 3a.

### 2.7. Determining the Concentration of Copper Ions by Using the ICP-MS

The CRS electrodes coated with distinctive epoxy coatings were subjected to electrochemical measurements in a 2 wt% of ACQ saline solution for 24 h. Due to the higher reactivity of iron compared to copper, when iron comes into contact with copper ions in the solution, the iron oxidizes while the copper ions are reduced to form copper. As a result, over the course of the 24 h EIS measurement, the CRS electrode gradually oxidized, and the copper ions existing in the testing solution chamber were progressively reduced. The schematic illustration is shown in Scheme 3b.

After the reaction, the solution was filtered, and the remaining copper ion concentration was measured using inductively coupled plasma mass spectrometry (ICP-MS). This technique involves introducing the sample ions into a mass spectrometer through a vacuum interface, where a mass analyzer separates ions by their mass-to-charge ratio. The detector then performs qualitative and quantitative analysis, allowing for the precise quantification of concentration of copper ions in the solution.

## 3. Results and Discussion

### 3.1. Characterization of AP by FTIR, NMR, and Mass Spectroscopy

**Figure 1:**
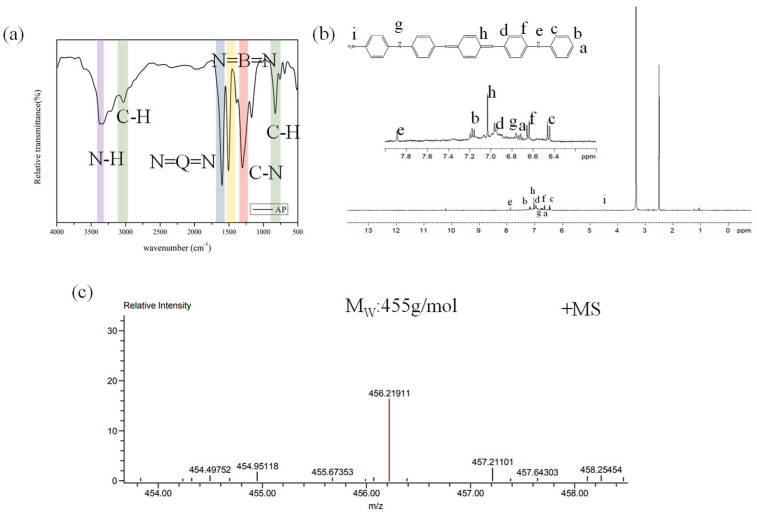
Characterization of as-synthesized aniline pentamer: (**a**) FT-IR spectrum, (**b**) 1H-NMR spectrum, and (**c**) Mass spectrum.

First, the as-synthesized AP was characterized by FTIR spectroscopy to identify its corresponding characteristic functional groups [42], as shown in Figure 1a. For example, the sharp peak at 824 cm^−1^ corresponds to the out-of-plane vibration of hydrogen on the benzene ring of AP. The peak signal at the position of 1304.6 cm^−1^ represents the C-N absorption of the amine group. The peaks located at 1507 cm^−1^ and 1598 cm^−1^ correspond to the C=C stretching vibrations in the benzene (N=B=N) and quinone rings (N=Q=N), respectively. Additionally, the broad peak at 3345 cm^−1^ indicates the N-H stretching absorption.

To further confirm- the chemical structure of as-synthesized AP, the proton nuclear magnetic resonance (^1^H-NMR) spectroscopy was used. Figure 1b shows the NMR spectrum of AP. In this, the chemical shifts at δ = 7.84 and δ = 6.76 ppm correspond to the signals of hydrogen atoms on the nitrogen of the aniline units, while the chemical shifts at δ = 7.17, 6.95, 6.65, and 6.45 ppm indicate the hydrogen on the benzene rings. The signal at δ = 7.02 ppm represents the hydrogen on the quinone ring.

To further confirm the molecular weight of as-synthesized AP, the UHPLC-qTOFMS was applied. As shown in Figure 1c, the mass-to-charge ratio corresponds to a molecular weight of 455.2 g/mol. The mass spectrometry result of +MS detected a value of 456.2 (M+H), verifying the successful synthesis of the AP.

### 3.2. Redox Capability of AP Determined by UV–Visible Spectroscopy and CV

**Figure 2:**
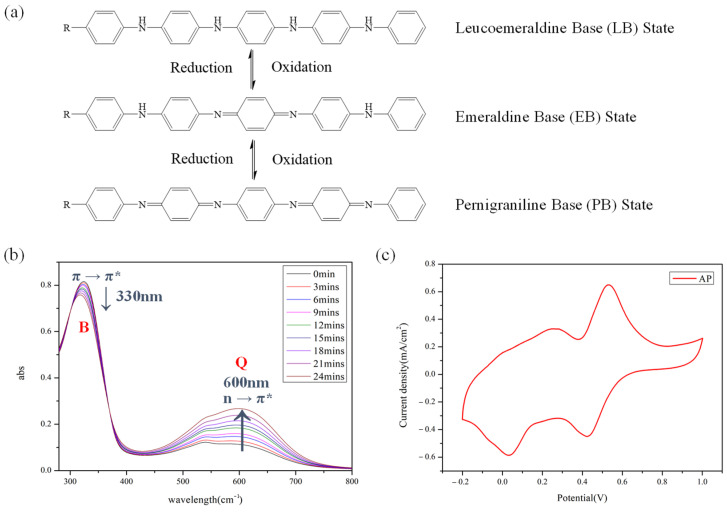
(**a**) Schematic diagram for the reversible redox behavior of aniline pentamer (AP); (**b**) Step-wisely chemical oxidation of AP by oxidant monitored using UV–vis absorption spectrophotometer. (**c**) Reversible redox capability of AP investigated by electrochemical cyclic voltammetry.

The AP was synthesized by oxidative coupling reaction between 4,4-diamino-diphenylaminesulfate hydrate and N,N-diphenyl-1,4-phenylenediamine. The as-synthesized AP exhibited three distinctive redox states, which is similar to that of polyaniline. Specifically, the synthesized AP is in the redox state of emeraldine base (EB) form, which is a state containing partially oxidized and partially reduced units, as shown in Figure 2a. The AP can be reduced to the fully reduced leuco-emeraldine base (LB) state or can be oxidized to fully oxidized state of pernigraniline base (PB), respectively, as shown at the top of Figure 2a.

This redox process of AP can be monitored by using UV–visible absorption spectroscopy. The lines with distinctive color (i.e., black, green, and brown) correspond to the fully reduced, half-oxidized, and fully oxidized states of as-synthesized AP, respectively, as shown in Figure 2b. The absorption peak appearing at wavelengths of 330 nm corresponds to the π→π* transition on the benzene ring of AP, while the absorption peak appearing at the wavelengths of 600 nm corresponds to the n→π* transition on the quinone ring of AP [42]. Before oxidation, the black line shows a weak quinone signal. However, after addition of oxidizing agent step-wisely, the quinone signal of AP becomes more pronounced gradually, as shown in Figure 2b. Continued oxidation shifts the quinone signal from wavelengths of 600 nm to 570 nm, with the brown line indicating the transition to the fully oxidized state of AP. 

In addition to analysis of UV–visible absorption spectroscopy, the electrochemical methods were also employed to examine the redox properties of AP. The scan voltage of AP was set from −0.2 V to 1.0 V at a scan rate of 100 mV/s. The redox curve of AP is shown in Figure 2c. It should be noted that the oxidation and reduction curve of AP exhibited two distinct peaks, individually. For example, the first oxidation peak appearing at the potential of 0.26 V indicates the transition from the fully reduced to the half-oxidized state, corresponding to the first electron transfer of AP. The second oxidation peak of AP appeared at the potential of 0.53 V, representing the transition from the half-oxidized to the fully oxidized state, indicating the second electron transfer of AP. On the other hand, two reduction peaks of AP appeared at the potential of 0.42 V and 0.03 V, representing the gradual reduction of AP back to its fully reduced state, completing the entire redox cycle. This electrochemical cyclic voltametric analysis further confirmed the distinct redox characteristics of as-synthesized electroactive AP.

### 3.3. Characterization of CTAB-Clay and AP-Clay by FTIR, XRD, and TGA

**Figure 3:**
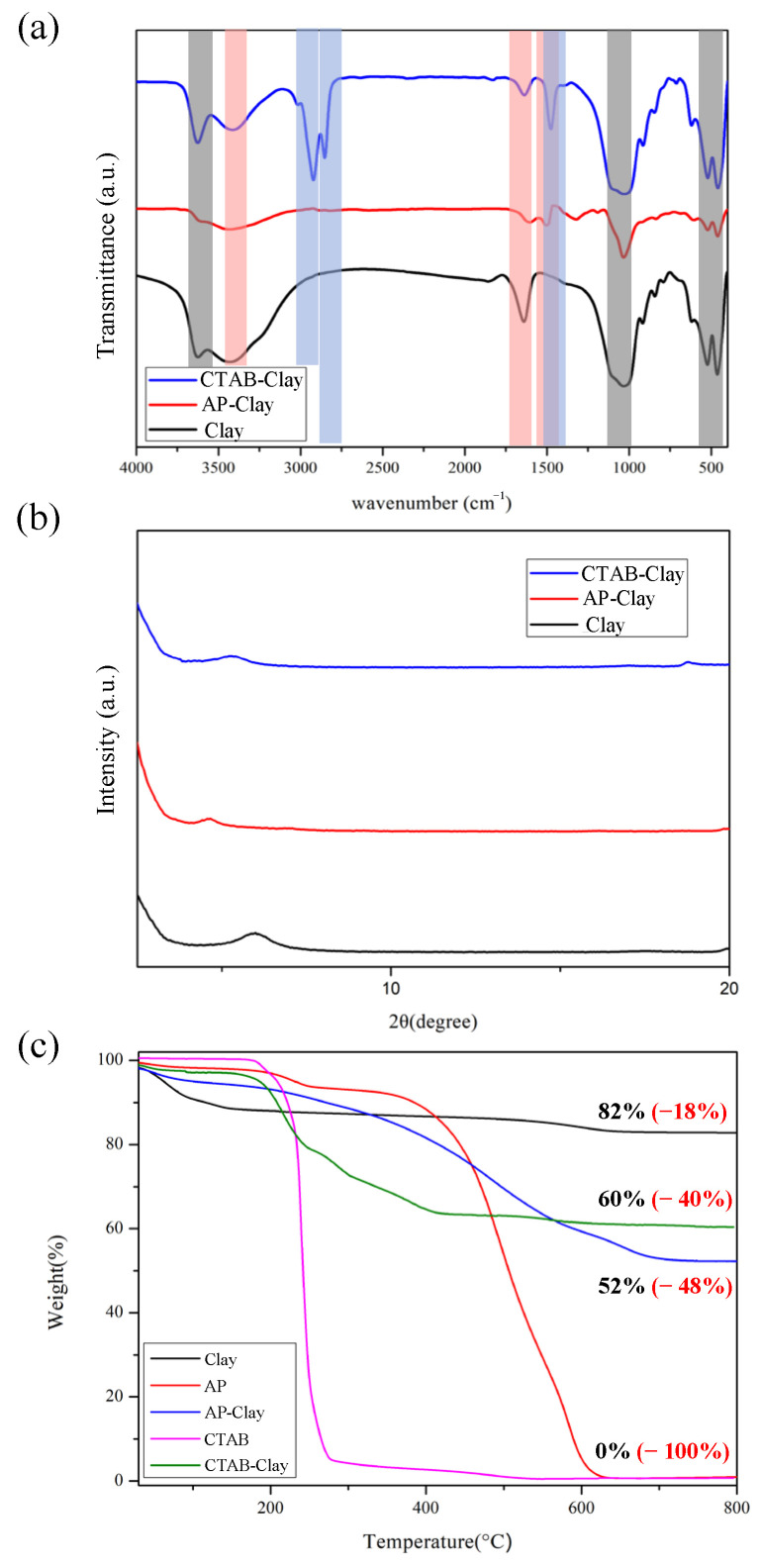
Characterization of organophilic clay of CTAB-clay and AP-clay by (**a**) FTIR, (**b**) XRD, and (**c**) TGA analysis.

In this study, FTIR spectroscopy was conducted to identify the characteristic functional groups of clay, CTAB-clay, and AP-clay. The FTIR spectra of commercial clay before and after modification with the intercalating agents of non-redox CTAB and redox AP is illustrated in Figure 3.

As shown in Figure 3a, the key functional group peaks of unmodified commercial clay, where the characteristic signals appeared at the wavenumber of 462 cm⁻^1^, 530 cm⁻^1^, and 1026 cm⁻^1^, correspond to the characteristic peaks of Mg-O, Al-O, and Si-O, respectively, of clay [19,20,21,22,23,24,25,26]. The FTIR spectrum of AP-clay exhibited the characteristic peaks of AP, in addition to the original clay peaks, indicating the successful modification of clay by AP through the cationic exchange reaction. Similarly, in the FTIR spectrum of CTAB-clay, alongside the original clay peaks, new absorption peaks of CTAB were observed at the wavenumber of 1470 cm⁻^1^, indicating C-N stretching, and also appeared at the wavenumbers of 2855 cm⁻^1^ and 2923 cm⁻^1^, corresponding to the C-H stretching vibrations of the surfactant [37]. These results concluded from the FTIR spectra confirmed the successful modification of clay by both intercalation agents of non-redox CTAB and redox AP.

On the other hand, the analysis of the XRD pattern was also used to evaluate the effect of OMC by observing the change of interlayer spacing of clay before and after the cationic exchange reaction between the Na^+^-MMT clay and the intercalation agents of CTAB and AP.

The interlayer spacing of clay, expressed as d-spacing, was calculated using Bragg’s equation: *nλ* = 2*d·*sinθ. As shown in Figure 3b, the unmodified commercial clay exhibited an initial *d*-spacing of 14.7 Å. After modification with AP, the interlayer spacing of OMC (i.e., AP-clay) was increased up to 18.9 Å. On the other hand, the interlayer spacing of OMC modified with CTAB was increased up to 16.8 Å. The results of XRD pattern indicated that both CTAB and AP can be intercalated successfully into the interlayer spacing of clay structure through the cationic exchange reaction. Moreover, the interlayer spacing of AP-clay was found to be obviously larger than that of CTAB-clay. This implied that AP-clay may exhibit a better dispersion capability in the epoxy matrix than CTAB-clay, which can be further confirmed by the TEM observation of as-prepared ECN materials.

Moreover, the thermal stability of as-prepared ECNs was further identified by thermogravimetric analysis (TGA). Figure 3c shows the thermal decomposition temperatures (T_d_) of the clay before and after organophilic modification. The T_d_ of unmodified commercial MMT clay was estimated to be 88 °C based on the 5 wt% of weight loss of the TGA curve. Moreover, the T_d_ of as-synthesized AP and CTAB was estimated to be 422 °C and 206 °C, respectively. After organophilic modification, the T_d_ of CTAB-clay and AP-clay was found to be increased up to 187 °C and 330 °C, respectively. This implied that the intercalation of both CTAB and AP into the interlayer spacing of clay may enhance the thermal stability of clay. Additionally, Figure 3c indicates that an increased quantity of residue remained in the samples after organophilic modification. First, the weight percentage of residue retained for unmodified commercial inorganic clay appearing at 700~800 °C was estimated to be ~82%. On the other hand, the small organic molecules of CTAB and AP were all decomposed after 300 °C and 600 °C, respectively. After organophilic modification, the residues of CTAB-clay and AP-clay were found to decrease to 60% and 52%, respectively.

### 3.4. Characterization and Properties of Epoxy–Clay Nanocomposites

**Figure 4:**
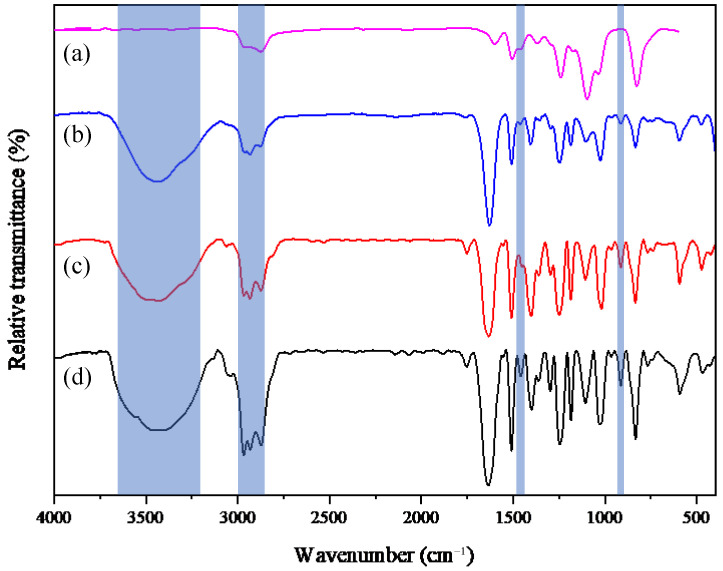
FTIR spectra of epoxy and corresponding epoxy–clay nanocomposites: (**a**) epoxy, (**b**) CTAB-clay-1, (**c**) AP-clay-1, and (**d**) AP-clay-5.

The FTIR spectra of a series of ECNs are shown in Figure 4. The ECN coatings containing 1 wt% and 5 wt% CTAB-clay and 1 and 5 wt% of AP-clay are denoted by CTAB-clay-1, CTAB-clay-5, AP-clay-1, and AP-clay-5, respectively. It should be noted that the characteristic peaks of CTAB-clay and AP-clay in ECN materials may be intensified as the loading of OMC was increased. The epoxide group of DGEBA was reacted with the amine group of T-403 by performing a thermal ring-opening polymerization reaction. After curing, the wavenumbers appearing at ~910 cm⁻^1^ (the epoxide absorption peak) and 3200 ~3500 cm⁻^1^ (the primary amine absorption peaks) disappeared, as shown in Figure 4a. This confirms the completeness of the thermal ring-opening polymerization reaction. The FTIR spectra for a series of ECN materials (i.e., CTAB-clay-1, AP-clay-1, and AP-clay-5) showed distinctive absorption bands at the wavenumbers of 1470 cm⁻^1^, 2855 cm⁻^1^, and 2923 cm⁻^1^, which corresponded to C-N and C-H stretching caused by the CTAB or AP-modified clay, as depicted in Figure 4b–d.

**Figure 5:**
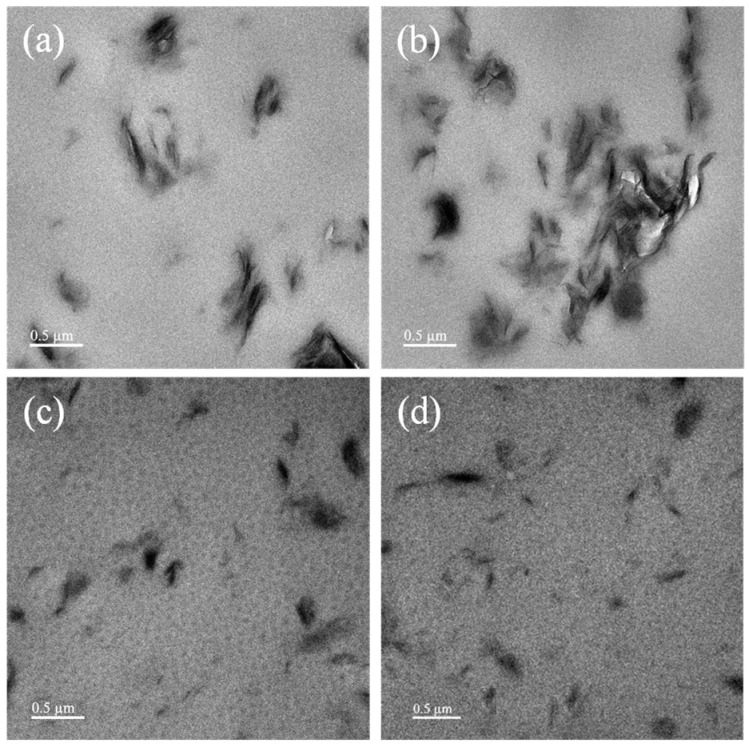
TEM images of a series of epoxy–clay intercalated nanocomposite coatings for (**a**) CTAB-clay-1, (**b**) CTAB-clay-5, (**c**) AP-clay-1, and (**d**) AP-clay-5.

On the other hand, in this study, the dispersion capability of OMC platelets (i.e., CTAB-clay and AP-clay) in epoxy matrix was observed by TEM at a magnification of 6000×, as shown in Figure 5. It should be noted that most of the OMC platelets exhibited an uneven dispersion/distribution within the epoxy matrix, indicating an intercalated dispersion state of OMC platelets in epoxy matrix. The CTAB-clay-5 nanocomposite exhibited obvious agglomeration. However, AP-clay-5 nanocomposite revealed no noticeable agglomeration. This suggested that the OMC platelets modified with the intercalation agent AP exhibited a better dispersion state compared to those modified by CTAB.

**Figure 6:**
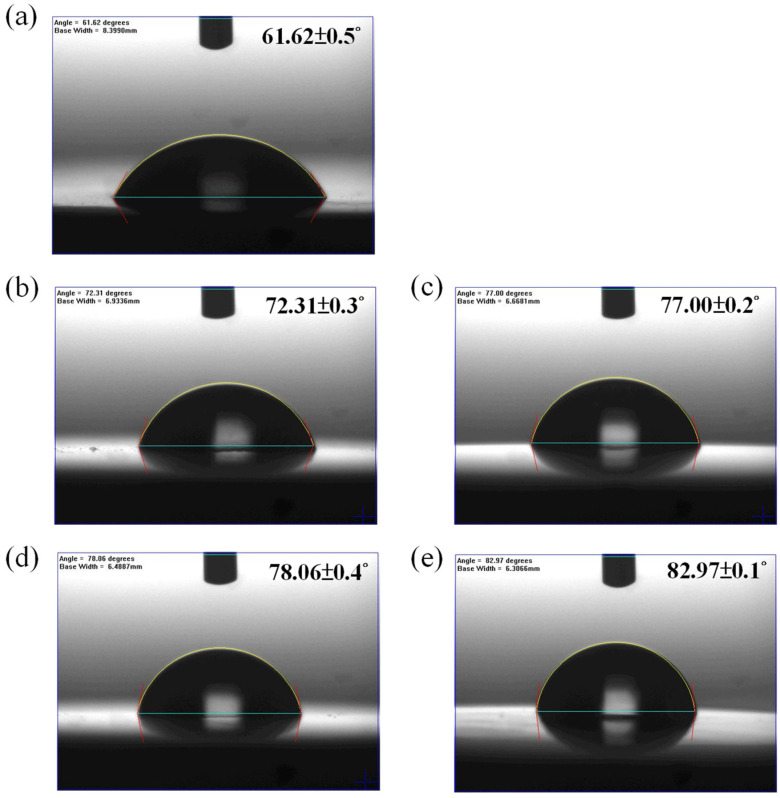
Surface wettability of coatings for (**a**) epoxy, (**b**) CTAB-clay-1, (**c**) CTAB-clay-5, (**d**) AP-clay-1, (**e**) AP-clay-5 determined by contact angle of water droplets.

To study the surface wettability of as-prepared ECN coatings, the contact angle (CA) of water droplets upon coatings was measured (Figure 6). The CA of neat epoxy coating was estimated to be ~61.62°, indicating that the surface of neat epoxy is hydrophilic. Moreover, by dispersion with 1 wt% of CTAB-clay and AP-clay, the CA of ECN coatings increased to ~72.31° and ~78.06°, respectively. As the loading of OMC platelets increased to 5 wt% of CTAB-clay and AP-clay, the CA of ECN materials also increased to ~77.00° and ~82.97°, respectively. In summary, the ECN coatings containing AP-clay exhibited a higher hydrophobicity than those containing CTAB-clay, which is more beneficial to the subsequent corrosion protection studies.

### 3.5. Electrochemical Impedance Spectroscopy Studies of ECN Coatings

**Figure 7:**
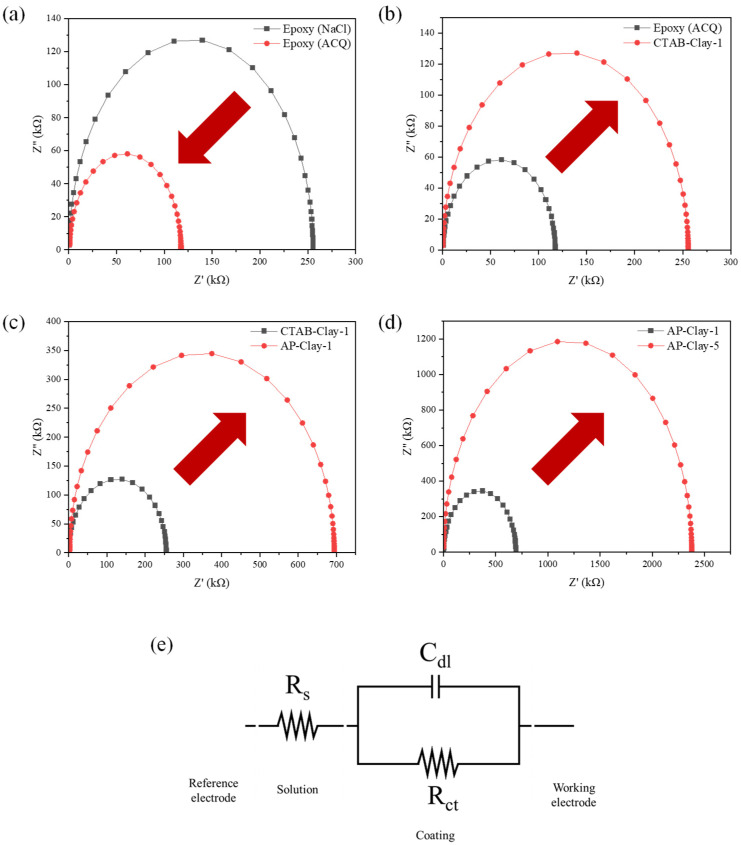
Comparative corrosion study based on the Nyquist plot of electrochemical impedance spectroscopy. (**a**) CRS electrode coated by epoxy immersed in the saline environment with/without ACQ, (**b**) CRS coated by epoxy coating with/without 1 wt% CTAB-clay immersed in saline environment with ACQ, (**c**) CRS coated by epoxy coating with 1 wt% of AP-clay and CTAB-clay immersed in saline environment with ACQ, (**d**) CRS coated by epoxy coating with 1 and 5 wt% AP-clay loading immersed in saline environment with ACQ, and (**e**) electrical equivalent-circuit models used for simulation of the impedance spectra of epoxy AP-clay coatings.

In this study, electrochemical impedance spectroscopy (EIS) was employed to investigate the corrosion resistance of the epoxy and derivative epoxy–clay nanocomposite coatings. Figure 7a shows the Nyquist plots for the neat epoxy coating immersed in saline environment (i.e., 3.5 wt% NaCl_(aq)_) without and with 2 wt% of ACQ wood preservatives. The impedance of neat epoxy coating immersed in the saline environment without ACQ was estimated to be ~255.5 kΩ·cm^2^. However, the impedance of neat epoxy coating immersed in saline environment with 2 wt% ACQ wood preservatives was found to decrease down to 121.3 kΩ·cm^2^, as shown in Figure 7a (Nyquist plot) and Table 1, indicating that the presence of ACQ may accelerate the corrosion rate of metal substrates. On the other hand, the impedance of neat epoxy coating and CTAB-clay-1 coating immersed in saline environment with 2 wt% ACQ was investigated, as shown in Figure 7b. For example, by dispersion of organophilic CTAB-clay, the impedance of epoxy coating was increased from 121.3 to 271.6 kΩ·cm^2^, reflecting that enhanced corrosion resistance of epoxy coating by incorporation of 1wt% of CTAB-clay. Moreover, the impedance of CTAB-clay-1 and AP-clay-1 coating immersed in saline environment with 2 wt% ACQ was investigated. The impedance of CTAB-clay-1 and AP-clay-1 coating was estimated to be ~271.6 and ~702.5 kΩ·cm^2^, respectively, as shown in Figure 7c and Table 1, reflecting that the ECN coating containing 1 wt% of AP-clay exhibited a more effective anticorrosion performance than that of CTAB-clay. The enhancement of corrosion protection of AP-clay-1 as compared to that of CTAB-clay-1 may be attributed to two possible reasons. First, OMC modified with AP may lead to a better dispersion as compared to that of CTAB, as evidenced by the TEM image of ECN materials. The better dispersion state of AP-clay may effectively increase the diffusion pathway of oxygen gas and copper ions (Cu^2+^) in ACQ solution, as evidenced by the following study of gas permeation analysis (GPA) and ICP-MS, respectively. Secondly, the redox capability of AP-clay appearing at the interface of coating and metallic substrates may induce the formation of a densely passive metal oxide layer to protect the underlying metal substrate, which can be evidenced by the following study of XRD patterns. In the study testing loading of hydrophilic redox AP-clay in ECN coatings, the impedance of AP-clay-1 and AP-clay-5 coating immersed in saline environment with 2 wt% ACQ was also investigated. For example, the impedance of AP-clay-1 and AP-clay-5 coating was estimated to be ~702.5 and ~2389 kΩ·cm^2^, respectively, as shown in Figure 7d and Table 1, indicating that higher loading of hydrophilic AP-clay in ECN coating results in better the corrosion protection performance.

The electrochemical behavior of the coatings was analyzed by fitting the Nyquist plots with an appropriate electrical equivalent circuit (EEC) model. The impedance spectra exhibited a typical single semicircle shape, indicating that the corrosion process was predominantly governed by charge transfer at the coating/solution interface. Accordingly, a simplified Randles-type circuit was employed, consisting of the solution resistance (R_s_) in series with a parallel combination of the double-layer capacitance (C_dl_) and the charge transfer resistance (R_ct_). In this model, Rs represents the ohmic resistance of the electrolyte solution, while C_dl_ accounts for the capacitive behavior associated with the electric double layer formed at the interface. The R_ct_ parameter reflects the resistance to charge transfer reactions occurring at the metal surface and is directly related to the corrosion protection performance of the coating. A larger semicircle diameter in the Nyquist plot corresponds to a higher R_ct_ value, signifying enhanced barrier properties and improved corrosion resistance.

### 3.6. Salt Spray Testing of ECN Coatings

**Figure 8:**
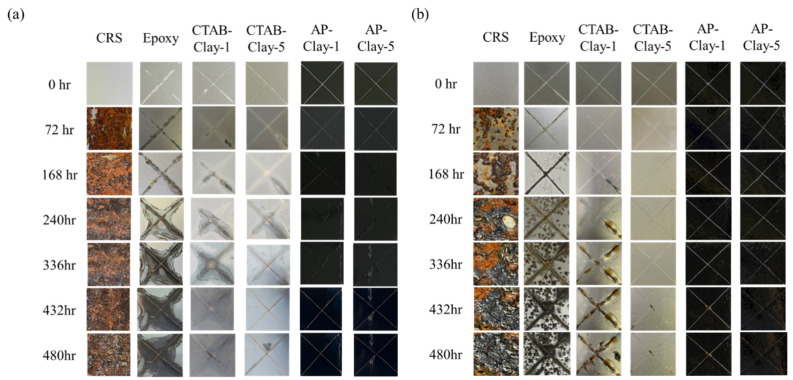
Salt spray test analysis based on ASTM B-117 of raw steel panel and steel panel coated with epoxy, CTAB-clay-1, CTAB-clay-5, AP-clay-1, AP-clay-5 in the saline environment (**a**) without ACQ and (**b**) with ACQ.

In accelerated salt-spray testing, six steel panels coated with five distinctive coatings (denoted by epoxy, CTAB-clay-1, CTAB-clay-5, AP-clay-1, and AP-clay-5) were used based on the ASTM B-117. After coating treatment, steel panels without or with coatings were scratched with the shape of an “X” on the surface, then placed in a standard salt-spray chamber. It should be noted that the photographs of the scratched areas of steel panel were taken at time intervals of 0, 72, 168, 240, 336, 432, and 480 h to visually observe the corrosion behavior of the samples. It can be observed clearly that the steel panels with/without coatings exhibited severe corrosion behavior in the salt-spray chamber with ACQ solution (Figure 8b) as compared to those without ACQ solution (Figure 8a).

In Figure 8a, after 72 h of salt-spray exposure with ACQ solution, noticeable corrosion can be observed on the steel panels coated with neat epoxy coating, as well as with CTAB-clay-1 and CTAB-clay-5. The steel panel coated with neat epoxy coating was found to exhibit quite severe corrosion, while those with CTAB-clay-1 and CTAB-clay-5 showed only slight signs of degradation. After 168 h of salt-spray testing, no noticeable corrosion behavior was observed on the center “X” of the steel panel coated with AP-clay-1, while intensified corrosion behavior was visually observed in the center “X” of the steel panels coated with neat epoxy, CTAB-clay-1, and CTAB-clay-5. Eventually, after 480 h of testing, there was still no noticeable corrosion behavior observed on the center “X” of steel panels coated with AP-clay-1 and AP-clay-5, while clearly intensified corrosion behavior was visually observed on the center “X” of steel panels coated with neat epoxy, CTAB-clay-1, and CTAB-clay-5. In Figure 8b, the steel panel coated with five distinctive epoxy coatings exposed in a salt-spray chamber containing ACQ solution also exhibited a similar trend. However, the corrosion behavior of the steel panels with/without epoxy coatings was significantly intense, which may be attributed to the copper ions existing in ACQ speeding up the corrosion rate of iron.

To sum up, three important inferences can be drawn as follows: (1) The steel panel coated with five distinctive epoxy coatings exposed in a salt-spray chamber with ACQ condition exhibited an intense corrosion behavior compared to the counterpart without ACQ. (2) After exposure in a salt-spray chamber, the steel panel coated by ECNs exhibited better corrosion protection as compared to that with neat epoxy coating. (3) After exposure in a salt-spray chamber, the steel panel coated with ECNs (i.e., AP-clay-1 and AP-clay-5) containing a redox intercalation agent was found to show a better corrosion protection performance compared to that coated by ECNs (i.e., CTAB-clay-1 and CTAB-clay-5) containing an un-redox intercalation agent. This was quite consistent with the previous EIS studies.

### 3.7. Determining the Concentrations of Copper Ions Before/After Electrochemical Corrosion Measurements by ICP-MS Spectra

Copper ions accelerate the corrosion of iron-based substrates primarily through galvanic and electrochemical mechanisms. When copper ions are present in a corrosive environment, they can deposit onto the iron surface, forming a cathodic layer that establishes a galvanic cell. This causes the underlying iron to act as the anode and corrode more rapidly. Additionally, the deposited copper enhances the oxygen reduction reaction, further driving the electrochemical corrosion process. This effect is particularly pronounced in environments containing chloride ions or during wet–dry cycles, where localized copper deposition can destabilize passive oxide films on iron and promote pitting.

When copper ions in solution come into contact with iron, the higher reactivity of iron compared to copper results in the oxidation reaction of iron and the reduction reaction of copper ions simultaneously, leading to the deposition of copper upon the iron surface. During a 24 h EIS measurement, the CRS electrode is progressively oxidized while copper ions are gradually deposited, as shown in Scheme 3b. We therefore determined the concentrations of copper ions before/after EIS measurements in a saline environment containing 2 wt% of ACQ by ICP-MS spectra.

**Figure 9:**
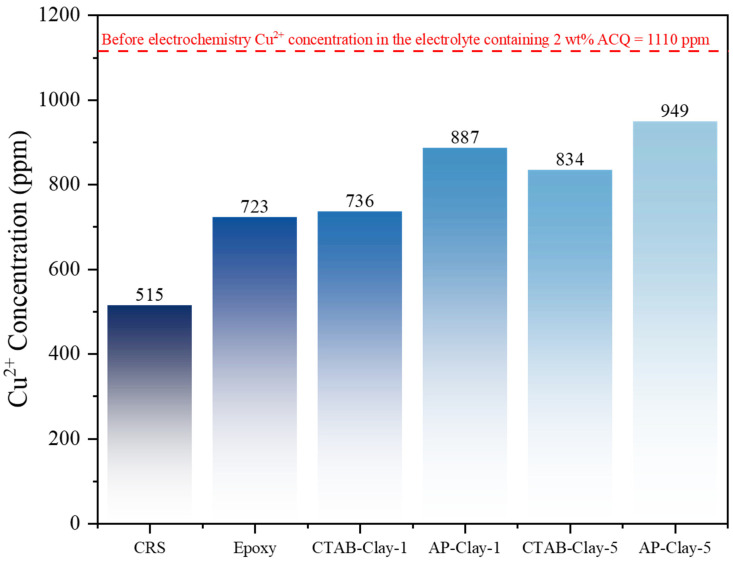
Determining the concentration of copper ions remaining in the testing chamber by ICP-MS after 24 h electrochemical impedance testing on the raw CRS, CRS-coated with five ECN coatings measured in saline environment containing 2wt% ACQ.

Before the EIS measurement, the CRS electrode was immersed in a saline environment containing 2 wt% of ACQ. The concentration of copper ions determined by ICP-MS was found to be ~1110 ppm. After the electrochemical EIS measurement of CRS electrodes for 24 h, the concentration of copper ions was found to be ~515 ppm, as shown in Figure 9, which indicated that after the EIS measurement, the concentration of copper ions decreased from 1110 ppm to 515 ppm. The decreased amount of concentrated copper ions was calculated to be ~595 ppm, which reduced the copper metal, reflecting that the surface of iron metals (i.e., CRS electrode) may be further oxidized to metal oxides.

Electrochemical impedance spectroscopy (EIS) conducted over a 24 h period revealed that copper ion concentrations in CRS samples coated with unmodified epoxy were reduced to approximately 723 ppm. This reflects a notable decrease from the initial level of 1110 ppm. Comparatively, a concentration drop of about 387 ppm was observed, which is significantly lower than the ~595 ppm typically detected in uncoated CRS electrodes. This reduction in copper ion levels is attributed to the barrier effect provided by the epoxy thermoset coating, which formed a physical obstruction roughly 150 μm in thickness on the substrate. As a result, the diffusion path for copper ions from the external surface to the epoxy/substrate interface was effectively extended, impeding ion migration into the metallic substrate.

On the other hand, the concentrations of copper ions for CRS coated with four different ECN coatings (i.e., CTAB-clay-1, AP-clay-1, CTAB-clay-5, and AP-clay-5) were found to be ~736, ~887, ~834, and ~949 ppm, respectively, as shown in Figure 9. It should be noted that, after 24 hEIS measurement, the four CRS panels coated with ECN coatings were found to exhibit a higher concentration of copper ions in the testing solution (i.e., saline environment containing 2 wt% of ACQ) than CRS panels coated with neat epoxy coating. This indicated that by dispersing the OMC into epoxy coating, the as-prepared ECN coatings may reveal a longer diffusion pathway for copper ions. Moreover, the concentration of copper ions of ECN containing organophilic AP-clay was higher than that of CTAB-clay, indicating that AP-clay may have a better dispersion state in epoxy matrix, therefore leading to a much longer diffusion pathway for copper ions than that of CTAB-clay in the order of: AP-clay-1 (~887 ppm) > CTAB-clay-1 (~736 ppm) or AP-clay-5 (~949 ppm) > CTAB-clay-5 (~834 ppm).

### 3.8. Gas Permeability Testing for Coatings

**Figure 10:**
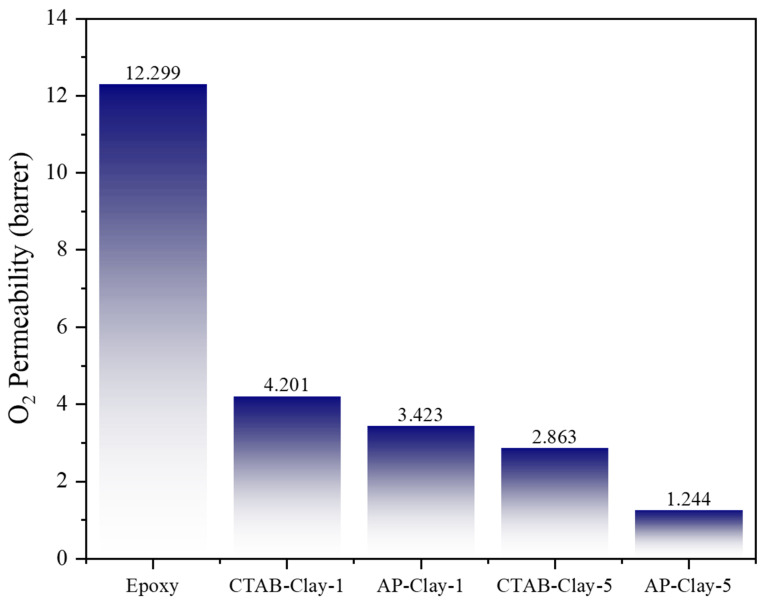
Permeability of oxygen gas for epoxy and corresponding four distinctive epoxy–clay nanocomposite free-standing film with a thickness of ~150 μm.

Corrosion inhibition of the CRS electrodes in ACQ-containing saline solutions arises from the combined effects of Cu^2^⁺ ions and dissolved oxygen in the electrolyte. As shown by ICP–MS (inductively coupled plasma-mass spectrometry) data in the previous section, the presence of Cu^2^⁺ in ACQ accelerates the anodic dissolution of iron. To evaluate the contribution of dissolved oxygen to corrosion, gas permeability analyzer (GPA) measurements were conducted on free-standing films of neat epoxy, CTAB-clay-1, AP-clay-1, CTAB-clay-5, and AP-clay-5 (Figure 10; Table 1). The oxygen permeability of a 150 µm-thick, neat epoxy free-standing film was approximately 12.299 barrer. Incorporation of 1 wt% CTAB-clay reduced O₂ permeability to ~4.201 barrer, while 1 wt% AP-clay yielded ~3.423 barrer. At 5 wt% clay loading, CTAB-clay-5 and AP-clay-5 films exhibited oxygen permeabilities of ~2.863 barrer and ~1.244 barrer, respectively. All ECN films displayed significantly lower O₂ permeability than the unmodified epoxy, indicating that organophilic clay platelets create a more tortuous diffusion path for oxygen molecules, thereby enhancing protection against oxygen-driven corrosion. Moreover, AP-clay consistently outperformed CTAB-clay at equivalent loadings—AP-clay-1 (3.423 barrer) < CTAB-clay-1 (4.201 barrer), and AP-clay-5 (1.244 barrer) < CTAB-clay-5 (2.863 barrer). Increasing clay content further improved barrier properties: CTAB-clay-5 (2.863 barrer) < CTAB-clay-1 (4.201 barrer) and AP-clay-5 (1.244 barrer) < AP-clay-1 (3.423 barrer). These results confirm that higher organophilic clay loading enhances resistance to oxygen permeation, contributing to the overall anticorrosive performance of the coatings.

### 3.9. Characterization of Densely Passive Metal Oxide Layer Induced by the Electro-Catalyzed/Redox Capability of Aniline Pentamer (AP) Existing in Organophilic Clay upon CRS Electrode by XRD

**Figure 11:**
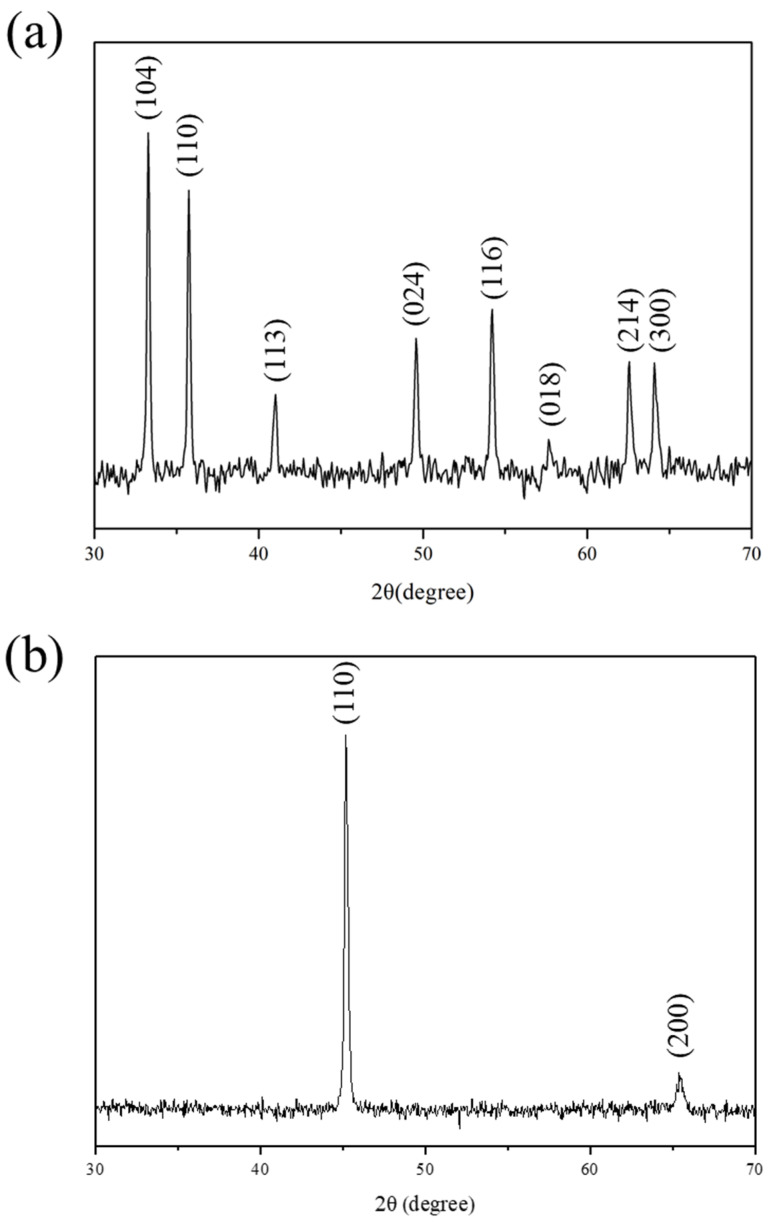
XRD pattern for the surface of (**a**) CRS electrode coated in AP-clay-5 for 30 days, followed by detaching the AP-clay-5 coating, and (**b**) CRS electrode coated in CTAB-clay-5 for 30 days, followed by detaching the CTAB-clay-5 coating.

The as-synthesized AP, similar to polyaniline, exhibited reversible redox capability, which had been identified by the previous study of electrochemical cyclic voltammetry and monitored by the UV–visible absorption spectroscopy. In this study, AP-clay-5 was used for the testing samples to study the formation of densely passive metal oxide layers upon the surface of CRS electrode. After electrochemical corrosion measurement, CRS electrodes coated with AP-clay-5 were employed as testing samples. First, the coating on the CRS electrodes was detached, then XRD was used to investigate the surface of the CRS electrodes. Figure 11a shows the XRD pattern on the surface of CRS electrodes after detaching the AP-clay-5 coating. It should be noted that eight distinctive characteristic peaks were observed at values of 2θ = 33.3°, 35.8°, 41.0°, 49.7°, 54.2°, 62.6°, and 64.1°, which may correspond to the α-Fe_2_O_3_ (hematite) rhombohedral lattice planes (104), (110), (113), (024), (116), (018), (214), and (300), as identified by comparison with JCPDS NO. 33-0664 [43]. The formation of the α-Fe_2_O_3_ on the CRS electrodes may have been induced by the electrocatalytic activity of AP present in the AP-clay at the interface between the ECN coating and the CRS electrodes. As a control experiment, a CRS electrode coated with CTAB-clay-5 was also investigated. However, as shown in Figure 11b, there were no characteristic peaks of α-Fe_2_O_3_ in the XRD pattern for the surface of the CRS electrode after detaching the CTAB-clay-5 coating (JCPDS NO. 3-1050).

### 3.10. Mechanisms of Enhanced Diffusion Resistance and Corrosion Protection via Clay Modification

The modification of clay plays a critical role in improving both diffusion resistance and corrosion protection of epoxy composite nanocoatings (ECNs). Organomodified clays (OMCs), such as those intercalated with CTAB or AP, exhibit a layered structure that, when well-dispersed in the epoxy matrix, forms a tortuous pathway that significantly impedes the diffusion of water, oxygen, and corrosive ions toward the metal substrate. This physical barrier effect slows down the transport of corrosive species, thereby enhancing protection. Additionally, surface modification improves the compatibility between clay and the organic epoxy matrix, preventing clay agglomeration and promoting uniform dispersion. In particular, the redox-active AP-clay not only contributes to a tighter interfacial interaction with the epoxy resin through hydrogen bonding or π–π interactions, but also introduces an active corrosion protection mechanism. The redox capability of AP facilitates the formation of a dense and stable α-Fe₂O₃ passive layer at the coating–metal interface, further reducing electron transfer and suppressing corrosion reactions. Therefore, clay modification enhances the performance of ECNs through both passive (barrier) and active (electrochemical) protection mechanisms, providing long-term durability in aggressive environments.

## 4. Conclusions

In this study, two types of organomodified clays (OMCs) were synthesized using non-redox cetyltrimethylammonium bromide (CTAB) and redox-active 4-aminophenol (AP) as intercalating agents, and incorporated into epoxy composite nanocoatings (ECNs) to evaluate their anticorrosion performance in saline environments containing 2 wt% ACQ wood preservatives. The AP intercalating agent was synthesized via oxidative coupling and confirmed by ^1^H-NMR, FTIR, and mass spectrometry, with its redox behavior verified through cyclic voltammetry and UV–vis spectroscopy. ECNs containing 1 wt% AP-clay exhibited the highest corrosion resistance, with an impedance of ~702.5 kΩ, compared to ~271.6 kΩ for CTAB-clay-1 and ~121.3 kΩ for neat epoxy with ACQ. The improved performance of AP-clay-1 is attributed to enhanced dispersion of clay platelets and the formation of protective α-Fe_2_O_3_ layers at the coating–metal interface due to the redox functionality of AP. These findings, supported by EIS and accelerated salt-spray tests, demonstrate that redox-active OMCs can significantly enhance the corrosion protection of epoxy coatings in aggressive environments.

## Figures and Tables

**Table 1:** Electrochemical impedance measurement, residual concentration of Cu^2+^, and oxygen permeability of raw CRS electrodes and CRS coated with distinctive epoxy coatings immersed in 3.5 wt% of NaCl aqueous solution containing 2 wt% of ACQ.

Sample Code	Material Composition				Electrochemical Impedance Measurements		Thickness (μm)	Concentration of Cu^2+^ (ppm)	Oxygen Permeability (barrer)
	DGEBA (wt%)	T-403 (wt%)	CTAB (wt%)	AP (wt%)	Z’ @NaCl (kΩ)	Z’ @2wt% ACQ (kΩ)			
CRS	-	-	-	-	0.049	0.036	-	515	-
Epoxy	71.4	28.6	-	-	255.5	121.3	150 ± 3	723	12.299
CTAB-clay-1	70.7	28.3	1	-	382.4	271.6	152 ± 2	736	4.201
AP-clay-1	70.7	28.3	-	1	779.3	702.5	151 ± 1	887	3.423
CTAB-clay-5	67.9	27.1	5	-	1050	905.5	149 ± 1	834	2.863
AP-clay-5	67.9	27.1	-	5	2770	2389	150 ± 1	949	1.244

## Data Availability

Data are contained within the article.
